# Identification of Novel *KMT2B* Variants in Chinese Dystonia Patients via Whole-Exome Sequencing

**DOI:** 10.3389/fneur.2019.00729

**Published:** 2019-07-04

**Authors:** Jun Ma, Lin Wang, Yingmai Yang, Shanglin Li, Xinhua Wan

**Affiliations:** ^1^Department of Neurology, Peking Union Medical College Hospital, Chinese Academy of Medical Sciences, Beijing, China; ^2^Department of Geriatrics, Qilu Hospital of Shandong University, Jinan, China

**Keywords:** dystonia, whole-exome sequencing, *KMT2B*, novel, Chinese

## Abstract

**Background:** Dystonia is a movement disorder with high clinical and genetic heterogeneity. Recently mutations in lysine-specific histone methyltransferase 2B (*KMT2B*) gene have been reported to be associated with early-onset progressive dystonia.

**Methods:** We performed whole-exome sequencings (WES) in a cohort of early-onset dystonia patients from China. Bioinformatics analysis and cosegregation testings were conducted to select candidate causal variants. The effects of identified variants were classified according to the American College of Medical Genetics and Genomics (ACMG) standards and guidelines.

**Results:** Three novel *KMT2B* variants were identified, including p.Q1359^*^ in patient 1, p.R1487AfsTer7 in patient 2, and p.R152W in patient 3. Among these variants, the nonsense variant p.Q1359^*^ and the frameshift variant p.R1487AfsTer7 showed high pathogenicity and were rated as pathogenic according to the ACMG guideline. Regarding the phenotypes of these two patients with pathogenic variants, patient 2 showed the similar presentation as reported whereas patient 1 seemly harbored the atypical presentations, including later onset age, atypical sites of onset and milder degree of dystonia.

**Conclusions:** We further report three dystonia patients with novel variants in *KMT2B* and expand the spectrums of genotype and phenotype of *KMT2B*.

## Introduction

Dystonia is known as a group of clinically and etiologically heterogeneous disorders characterized by sustained or intermittent muscle contractions causing abnormal, often repetitive, movements, postures, or both (1). Genetically defective components play an important role in the genesis of dystonia. With the advent of next-generation sequencing (NGS) technology, an expanding spectrum of dystonia associated genes have been identified (2). In 2016, two groups independently reported mutations in a newly identified gene, lysine-specific histone methyltransferase 2B (*KMT2B*) in patients with early-onset generalized dystonia (3, 4). Till now, more than 40 patients with different *KMT2B* variants have been reported, among which only one is from China (3–13). In this study, we screened *KMT2B* in a cohort of early-onset Chinese dystonia patients by whole-exome sequencing to expand the current knowledge on this gene.

## Methods

### Subjects

This study was carried out in a cohort of 52 unrelated dystonia patients (29 females, 23 males) from the Movement Disorders Clinic in the Department of Neurology, Peking Union Medical College Hospital in China. Detailed demographic and clinical characteristics of all recruited patients were summarized in the Table S1. All these patients were required to be diagnosed as dystonia with onset of dystonia before 26 years old (the cut-off age of early-onset defined by Chinese dystonia guideline). The mean age at onset was 15.7 years ranging from 1 to 26. Among these patients, 18 had focal dystonia, 10 had segmental dystonia, 12 had multifocal dystonia, 11 had generalized dystonia, and 1 had paroxysmal dystonia. Patients who were suspected of acquired etiologies were excluded. Most patients (43/52, 82.7%) were categorized as isolated dystonia, whereas nine patients manifested with additional symptoms including myoclonus (2/9), cognitive impairment (3/9), parkinsonism (3/9), chorea(1/9), epilepsy (1/9), and impaired vision (1/9). The study was approved by the ethics committee of Peking Union Medical College Hospital. Written informed consents were obtained from all patients or their legal guardians.

### Genetic Analysis

Genomic DNAs were extracted from peripheral blood samples of all the patients. After exclusion of the two most common genes (*TOR1A, THAP1*) for dystonia by Sanger sequencing, whole-exome sequencings (WES) were carried out in all patients. The detailed methods for WES were shown in Supplementary Materials. Briefly, variants were called, aligned, annotated and filtered. Only missense, nonsense, splice-site, in-frame insertion/deletion and frameshift variants with population minor allele frequency (MAF) <1% in public databases of normal human variation were selected for further assessment. The referenced public databases included 1000 Genomes Project (http://www.ncbi.nlm.nih.gov/variation/tools/1000genomes/), Exome Aggregation Consortium (ExAC; http://exac.broadinstitute.org/) and genome Aggregation Database (genomAD, http://gnomad-old.broadinstitute.org/). Pathogenicity prediction were performed by SIFT (http://sift.jcvi.org), PolyPhen-2 (http://genetics.bwh.harvard.edu/pph2), MutationTaster (http://www.mutationtaster.org), and Combined Annotation Dependent Depletion (CADD; http://cadd.gs.washington.edu). Potential causal variants were further verified by Sanger sequencing and tested for cosegregation in their available family members. Then these variants were tested in 100 Chinese unrelated healthy individuals by Sanger sequencing. The clinical effects of identified variants were classified according to the American College of Medical Genetics and Genomics (ACMG) standards and guidelines (14).

## Results

In this study, three novel *KMT2B* (NM_014727.2) variants were detected, including one nonsense variant c.4075C>T (p.Q1359^*^) in patient 1, one frameshift variant c.4458delC (p.R1487AfsTer7) in patient 2, and one missense variant c.454C>T (p.R152W) in patient 3 (Table 1). The clinical features of affected patients were summarized in Table 2.

**Table 1:** Summary of *KMT2B* novel variants identified in three probands with dystonia.

**Patient no**.	**cDNA**	**Protein**	**Variant type**	**Exon**	**Inheritance**	**1000 Genomes**	**ExAc**	**gnomAD**	**PolyPhen2 (score)**	**SIFT (score)**	**Mutation Taster**	**CADD**
1	c.4075C>T	p.Q1359*	Nonsense	15	*De novo*	Not found	Not found	Not found	NA	NA	Disease causing	38
2	c.4458delC	p.R1487AfsTer7	Frameshift	18	*De novo*	Not found	Not found	Not found	NA	NA	NA	29.5
3	c.454C>T	p.R152W	Missense	3	Paternal	0.000599042	0.0002531	0.0002246	Probable damaging (0.990)	Deleterious (0)	Disease causing	25.7

ExAC, exome aggregation consortium; PolyPhen2, Polymorphism Phenotyping v2; SIFT, Sorting intolerant from tolerant; CADD, Combined annotation dependent depletion; gnomAD, genome Aggregation Database; NA, not available.

*KMT2B reference sequence used: NM_014727.2*.

**Table 2:** Clinical findings of patients with *KMT2B* variants in this study.

**Patient**	**Patient 1**	**Patient 2**	**Patient 3**
Genotype	p.Q1359*	p.R1487AfsTer7	p.R152W
Age/Sex	31/M	15/F	33/F
Familial history	No	No	No
Age at onset	24	7	18
Site of onset	Larynx	Feet	Right hand
Involuntary movements	Dystonia	Dystonia	Dystonia
Distribution of dystonia	Left leg, larynx	Feet, right hand, larynx	Neck, trunk, right hand
Other features	Short statue, microcephaly, and bulbous nasal tip	No	No
Brain MRI	Normal	Normal	Normal

### Patient 1

**Figure 1:**
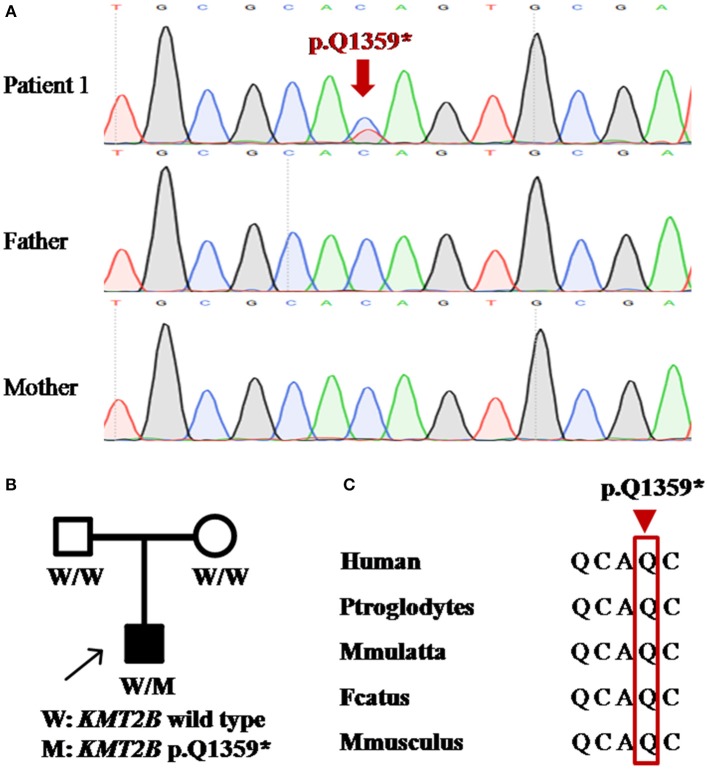
Detection of *KMT2B* nonsense mutation p.Q1359* in patient 1. **(A)** DNA sequencing chromatograms of portions of *KMT2B* gene nonsense mutation (p.Q1359*) (red arrowed). **(B)** Pedigree chart of patient 1. **(A,B)** demonstrated the *de novo* status of the nonsense mutation. **(C)** Conservation across multiple species at position 1359 (red rectangle).

Patient 1 was a 31 year-old man who was the only child born to his healthy non-consanguineous parents. Family history and delivery history of the patient were unremarkable. At the age of 24, he first developed slurred speech. The symptom progressed slowly. Mild dystonia of left leg were later noticed when he was admitted to our hospital (Video S1). Also, short statue, microcephaly and bulbous nasal tip were present in this patient. However, there was no evidence of additional neurological features such as developmental delay, intellectual disability and seizure. Brain magnetic resonance imaging (MRI) was unremarkable from the age of 24 to 31. Administration of medication, including levodopa, baclofen and benzhexol, did not show any clinical benefit. Whole-exome sequencing (WES) was performed and detected a novel heterozygous stop-gain variant c.4075C>T (p.Q1359^*^) in *KMT2B*. The variant was absent in dbSNP, ExAc, 1000 Genomes, and gnomAD. It was predicted to “disease causing” by MutationTaster and scored 38 by CADD. Segregation analysis revealed that the stop-gain variant was absent in his parents which indicated it was *de novo* (Table 1; Figure 1).

### Patient 2

**Figure 2:**
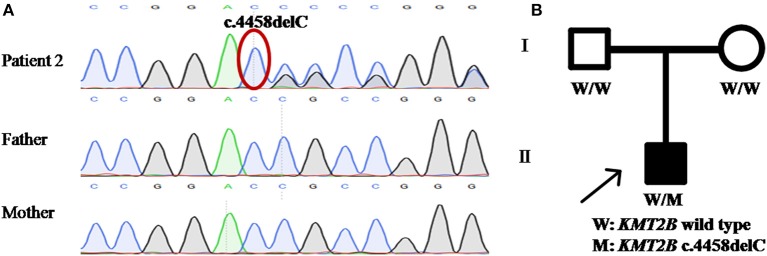
Detection of *KMT2B* frameshift variant p.R1487AfsTer7 in patient 2. **(A)** DNA sequencing chromatograms of portions of *KMT2B* gene frameshift variant p.R1487AfsTer7 (red circle). **(B)** Pedigree chart of patient 2. **(A,B)** demonstrated the *de novo* status of the frameshift variant.

Patient 2 was a 15 year-old girl from a non-consanguineous family with negative family history. She firstly experienced abnormal gait with dystonia posturing in her feet at the age of 7 years. During the following years, she developed slurred speech, followed by abnormal posture of right hand, leading to handwriting difficulty (Video S2). The severity of these symptoms mildly progressed, especially the severe dysphonia. Neuroimaging of brain showed no obvious abnormalities. Medical interventions including levodopa were of little effect. The testings of gene *GCH1, TOR1A*, and *THAP1* did not detect any pathogenic variants. WES was then performed and uncovered a heterozygous *KMT2B* frameshift variant c.4458delC (p.R1487AfsTer7), which was never reported before. This variant was absent in dbSNP, ExAc, 1000 Genomes and gnomAD, and predicted a score of 29.5 by CADD. Segregation analysis demonstrated the *de novo* status of the variant (Table 1; Figure 2).

### Patient 3

Patient 3 was a 33 year-old woman, who first developed right-handed writer's cramp at 18 years old. The symptom was improved after the treatment of botulinum toxin A injection. At 28 years old, a remarkable spasmodic torticollis was developed and progressively deteriorated in the following years. On current examination, in addition to the demonstrated cervical dystonia, abnormal posture of trunk was also noted (Video S3). In this patient, exome sequencing uncovered a novel heterozygous missense variant c.454C>T (p.R152W) in *KMT2B*, which was predicted to be damaging by SIFT, PolyPhen2, and MutationTaster. The CADD score was 25.7. The variant was rare in ExAc, 1000 Genomes and gnomAD (MAF 0.02531%, 0.0599042%, and 0.02246%, respectively). Segregation analysis revealed that the variant was inherited from her father who did not show any abnormal symptoms at current examination (Table 1; Figure S1).

In addition, several variants in other dystonia related genes were also identified, including *GCH1* c.638_641del in patient 4, *PINK1* c.1474C>T and c.938C>T in patient 7, *SGCE* c.304C>T in patient 26, *VPS13A* c.7867C>T in patient 23, and *ANO3* c.970A>G in patient 40.

## Discussion

*KMT2B* is a large gene (NM_014727.2: 37 exons, 8,148 bp), encoding a lysine methlytransferase specifically responsible for the methylation of histone H3 at lysine 4 (H3K4), an important epigenetic modification associated with active gene transcription (4). Its mutations have been recently reported to cause early-onset generalized dystonia (3, 4). Up to now, more than 40 patients with different *KMT2B* variants have been reported, including cases with interstitial microdeletion encompassing the entire gene, as well as cases with pathogenic variants (frameshift, in-frame deletion, splice-site, nonsense and missense variants) (3–11). For the majority of patients, *KMT2B* variants were confirmed as de novo, but autosomal dominant inheritance with reduced penetrance was also reported (4).

In this study, we found one nonsense mutation, one frameshift mutation and one missense mutation in three dystonia patients, among which c.4075C>T (p.Q1359^*^) was present in patient 1, c.4458delC (p.R1487AfsTer7) in patient 2, and c.454C>T (p.R152W) in patient 3. According to the criteria published by the ACMG (14), the variant c.4075C>T (p.Q1359^*^) in patient 1 was rated as “pathogenic” because this variant was a null variant in *KMT2B* [very strong pathogenic criterion (PVS)], proven to be *de novo* in origin [strong pathogenic criterion 2 (PS2)], absent from population databases [moderate pathogenic criterion 2 (PM2)], and predicted to be deleterious by multiple computational methods [supporting pathogenic criterion 3 (PP3)]. Of note, this nonsense variant occurred within the functionally important PHD domain (4). Similar to above, the frameshift variant c.4458delC in patient 2 should also be categorized to be “pathogenic,” because it belonged to PVS, PS2, PM2, and PP3. In contrast, the missense variant c.454C>T in patient 3 was of uncertain significance because it only met the criteria PP3 (*In silico* analysis supporting a deleterious effect) according to the consensus of ACMG. The variant was not located in any functional domains of KMT2B protein. Notably, co-segregations of this patient were difficult to judge because of the possible incomplete penetrance of *KMT2B*. Consequently, further functional verification of this missense variant may be helpful.

Regarding the phenotypes, patients with *KMT2B* variants present a relatively similar disease course progressively evolving from childhood-onset lower-limb dystonia into generalized dystonia with prominent bulbar and craniocervical involvement (3, 4). In our study, between the patients with pathogenic *KMT2B* variants, patient 2 showed the similar presentations as reported whereas patient 1 seemly harbored the atypical presentations. Firstly, patient 1 had a later onset age (24 years) than the typical *KMT2B*-mutated patients (5.8 years in the original report) (3, 4). Secondly, the site of onset with larynx was also atypical because lower-limb dystonia was the initial symptom in the majority patients. What's more, as the core feature, the symptoms of dystonia in this patient were seemly much milder than previously reported patients. As reported, in addition to dystonia, other features were present to varying degrees in the majority of patients, including developmental delay, intellectual disability, oculomotor disturbances, microcephaly, and dysmorphic features (such as elongated face, bulbous nasal tip, and short stature) (4). In our study, patient 1 showed short statue, microcephaly and bulbous nasal tip in addition to dystonia, whereas patient 2 presented with isolated dystonia. Finally, symmetrical hypointensity of the globus pallidi on brain MRI were observed in some reported cases (4). However, no abnormal neuroimaging findings were showed in our patients. In addition, patient 3 also presented with atypical manifestations, including a later onset age (18 years old), atypical site of onset (right hand) and milder degree of dystonia. However, the meaning of the phenotype is unclear concerning the uncertainty of the pathogenicity of R152W.

With the development of sequencing technology, more and more atypical cases with *KMT2B* mutations have been reported, such as paroxysmal cervical dystonia, or isolated oromandibular dystonia, or global development delay without any evidence of dystonia (4, 9). Thus, clinically heterogeneous phenotypes bring great challenge on precise diagnosis of dystonia. This study suggests that performing WES on affected individuals is an effective method for mapping genes of patients with possible genetic cause.

In conclusion, we report three dystonia patients with novel variants in *KMT2B* and broaden the spectrums of genotype and phenotype of *KMT2B*. Among these variants, the novel nonsense variant c.4075C>T (p.Q1359^*^) and the frameshift variant c.4458delC (p.R1487AfsTer7) show high pathogenicity whereas the missense variant need to further verify.

## Data Availability

The sequencing data in our manuscript has been uploaded to SRA (Sequence Read Archive) of NCBI. The SRA accession is PRJNA549023. It will be accessible with the following link after the indicated release date: https://www.ncbi.nlm.nih.gov/sra/PRJNA549023.

## Ethics Statement

The study was approved by the ethics committee of Peking Union Medical College Hospital. Written informed consents were obtained from all patients or their legal guardians in accordance with the Declaration of Helsinki.

## Author Contributions

JM: conception of the work, data acquisition, statistical analysis, and writing of the first draft. XW: design and organization of the work, manuscript review, and critique. LW, YY, and SL: data acquisition, manuscript review, and critique.

### Conflict of Interest Statement

The authors declare that the research was conducted in the absence of any commercial or financial relationships that could be construed as a potential conflict of interest.
